# Laparoscopic Indocyanine Green Sentinel Lymph Node Mapping in Endometrial Cancer

**DOI:** 10.1245/s10434-016-5090-x

**Published:** 2016-01-20

**Authors:** Andrea Papadia, Sara Imboden, Franziska Siegenthaler, Maria Luisa Gasparri, Stefan Mohr, Susanne Lanz, Michael D. Mueller

**Affiliations:** Department of Obstetrics and Gynecology, University Hospital of Berne and University of Berne, Berne, Switzerland; Department of Gynecology and Obstetrics, “Sapienza” University of Rome, Rome, Italy

## Abstract

**Background:**

In endometrial cancer (EMCA), indocyanine green (ICG) sentinel lymph node (SLN) mapping has been reported, mainly in conjunction with robotic surgery.

**Objective:**

We aimed to evaluate detection rates, sensitivity, and false negative (FN) rate of laparoscopic ICG SLN mapping in EMCA, and to evaluate differences in surgical outcomes between patients subjected to SLN biopsy only versus lymphadenectomy.

**Methods:**

A retrospective analysis of EMCA patients undergoing ICG SLN mapping ± pelvic (PLND) and/or para-aortic lymphadenectomy (PALND) was performed. Detection rates were calculated for the entire cohort. Sensitivity and FN rates were calculated for patients undergoing lymphadenectomy after SLN mapping, and surgical outcome was compared among patients undergoing SLN mapping only versus lymphadenectomy.

**Results:**

Of 75 patients, 33 underwent SLN mapping and 42 underwent SLN mapping followed by PLND/PALND. Overall and bilateral detection rates were 96 % (72/75) and 88 % (66/75), respectively, and the median number of removed SLNs, pelvic non-SLNs (NSLN) and para-aortic NSLNs was 3, 27, and 19, respectively. With a FN rate of 8.3 %, only one patient had bilateral FN SLNs and a metastatic para-aortal NSLN. Estimated blood loss (EBL) and operative (OR) time were significantly lower in patients undergoing SLN mapping only. No differences in complication rates between patients undergoing SLN mapping only and patients undergoing lymphadenectomy were recorded.

**Conclusions:**

Laparoscopic ICG SLN mapping has excellent overall and bilateral detection rates and a low FN rate. Compared with lymphadenectomy, SLN biopsy is associated with significantly lower EBL and shorter OR time.

Since its definition in 1988, surgical staging in endometrial cancer (EMCA) has been controversial.^1^ Given the low risk of lymph node metastases in early-stage disease, it is believed that performing a pelvic (PLND) and para-aortic lymphadenectomy (PALND) in every patient is harmful rather than helpful. Various methods have been investigated to identify which patient benefit the most from surgical staging. The most widespread method to triage patients to surgical staging is via intraoperative identification of pathologic risk factors at frozen section 2–6; however, frozen section is limited in that it provides only the risk of lymph node metastases. Thus, in patients intraoperatively identified as low risk, no information on lymph node status has been gathered.

Recently, interest has shifted from an intraoperative evaluation and an all-or-nothing approach to sentinel lymph node (SLN) mapping.^7^,^8^ Although SLN mapping is well-established in other tumors, in National Comprehensive Cancer Network (NCCN) guidelines its application in EMCA was only recognized to be appropriate in 2014.^9^ In gynecological cancers, SLN mapping with ^99^Tc, blue dye, and indocyanine green (ICG), in conjunction with a near-infrared (NIR) fluorescence imaging system, has been reported.^10^–^12^ ICG seems to stand out over the other tracers with regard to detection rates.^13^,^14^ In gynecologic oncology, ICG SLN mapping has been reported, mainly in conjunction with a robotic platform.^12^,^13^ In the laparoscopic setting, its use has been reported by only two other groups in patients with endometrial and cervical cancer, and by our group in cervical cancer.^14^–^18^

The aim of this study was to evaluate overall and bilateral detection rates, sensitivity, and false negative (FN) rates of the laparoscopic ICG SLN mapping in EMCA. Furthermore, we sought to evaluate differences in surgical outcome between patients subjected to SLN biopsy only versus full lymphadenectomy.

## Materials and Methods

A retrospective analysis of all patients with EMCA or complex atypical hyperplasia (CAH) undergoing ICG SLN mapping at our Institution between December 2012 and September 2015 was performed. Data on SLN mapping were collected prospectively. The study was approved by the Institutional Review Board, and all patients signed informed consent.

Patients underwent SLN mapping followed by laparoscopic lymph node biopsy, total hysterectomy (TLH) and bilateral salpingo-oophorectomy (BSO). Routine preoperative work-up included a transvaginal sonogram, as well as a chest x-ray for low-risk tumors and a computed tomography (CT) scan for high-risk tumors. Focused imaging was performed based on clinical suspicion, and SLN mapping was performed with intraoperative cervical ICG injection. Following a diagnostic laparoscopy, the cervix was injected submucosally, 1 cm deep in the stroma at the four cardinal points, with a total of 8 ml of ICG. One vial of 25 mg ICG powder (Pulsion^®^) was previously suspended with 5 ml of sterile water. The fluorescent signal was then identified under NIR mode, and the SLNs excised and sent to permanent pathology. Frozen section analysis on the SLN was performed only in cases of suspicion for metastasis. Suspicious non-SLNs (NSLNs) were removed and sent for frozen section. At final histopathological analysis, a complete ultrastaging was performed in all cases [three slides of hematoxylin and eosin (H&E) 200 um, immunohistochemistry (IHC) if negative]. After having completed the TLH, the uterus was sent to frozen section and, based on identification of tumor risk factors and clinical judgment, a laparoscopic PLND and/or PALND was performed. In our institution, patients with type 2 EMCA, or with type 1 poorly differentiated and deeply invasive tumors, undergo a PPALND. Patients with type 1 well-differentiated lesions confined to the inner half of the myometrium undergo a SLN biopsy only. All other patients undergo a PLND.

Clinicopathologic characteristics were evaluated using the basic descriptive statistics. Overall and bilateral detection rates, sensitivity, and FN rate of the SLN mapping were calculated using Fisher’s exact test. The false positive rate was defined as zero. The overall detection rate was calculated by dividing the number of procedures in which at least one SLN was identified by the total number of procedures performed, and the bilateral detection rate was calculated by dividing the number of procedures in which at least one SLN was identified on each side of the pelvis by the total number of procedures performed. A true positive SLN was defined as a positive SLN identified with histopathological techniques, independent of regional lymph node status, while an FN SLN mapping was defined as a bilateral negative SLN in combination with a metastatic NSLN. The FN rate of the SLN mapping was calculated for patients who underwent at least a PLND after the SLN biopsy. Surgical data, including estimated blood loss (EBL), operative (OR) time, and intra- and postoperative complication rates, were compared among the group of patients undergoing SLN mapping only and those undergoing a full lymphadenectomy after the SLN biopsy, using the unpaired *t* test. To reduce the bias possibly linked to additional radical procedures, patients undergoing radical hysterectomy (RLH), radical colpectomy, or other non-EMCA concomitant-related surgeries were excluded from this analysis. Statistical analyses were performed using the R software (version 3.1.0). All *p* values were two-sided, and *p* values <0.05 were considered statistically significant.

## Results

Seventy-five patients were included in the study; their clinicopathologic characteristics are summarized in Table 1.

**Table 1:** Clinicopathologic characteristics of patients

Patients (*N* = 75)	
Median age, years (range)	65 (38–89)
Median BMI (range)	27.2 (17.4–47)
FIGO stage	
0 (intraepithelial neoplasia, complex hyperplasia)	2 (2.7)
I	53 (70.7)
II	5 (6.6)
III	14 (18.7)
IV	1 (1.3)
Histology	
Complex atypical hyplerplasia/intraepithelial neoplasia	2 (2.7)
Endometrioid	66 (88.0)
Serous	2 (2.7)
Clear cell	1 (1.3)
Carcinosarcoma	4 (5.3)
Grade	
1	24 (32.9)
2	30 (41.1)
3	19 (26.0)
Lymphovascular space invasion	
Present	17 (22.7)
Absent	58 (77.3)
Myometrial invasion	
Absent	2 (2.7)
<50 %	46 (61.3)
>50 %	27 (36.0)
Patients with lymph node metastases	11 (14.7)
Surgical lymph node assessment	
SLN biopsy only	33 (44.0)
SLN biopsy + PLND	13 (17.3)
SLN biopsy + PLND + PALND	29 (38.7)
Median number of PLNs (range)	27 (10–48)
Median number of PALNDs (range)	19 (9–56)
Operations performed	
Hysterectomy + BSO	33 (44.0)
Hysterectomy + BSO + PLND	13 (17.4)
Hysterectomy + BSO + PLND + PALND	26 (34.7)
Radical hysterectomy + radical colpectomy + BSO + PLND + PALND	1 (1.3)
Radical hysterectomy + hemicolectomy + BSO + PLND + PALND	1 (1.3)
Radical hysterectomy + BSO + PLND + PALND	1 (1.3)
+Laparotomy	1 (1.3)
+Omentectomy	2 (2.6)

Data are expressed as *n* (%) unless otherwise specified

*BMI* body mass index, *FIGO* International Federation of Gynecology and Obstetrics, *SLN* sentinel lymph node, *PLND* pelvic lymph node dissection, *PALND* para-aortic lymph node dissection, *BSO* bilateral salpingo-oophorectomy, *PLNs* pelvic lymph nodes

A TLH/BSO and SLN biopsy was performed on 33 patients. Of the remaining 42 patients, 13 underwent a PLND and 29 underwent a PPALND. Of the patients undergoing a full retroperitoneal staging, two also underwent an omentectomy. Three patients underwent an RLH due to suspicion of parametrial invasion on preoperative MRI in two cases, and vaginal involvement requiring a colpectomy in one case; one of these patients underwent a hemicolectomy for a concomitant colon cancer. All patients undergoing an RLH underwent a PPALND after the SLN biopsy. Conversion to laparotomy occurred in one case, secondary to macroscopic metastatic lymph node involvement. The median OR time was 220 min, and the median EBL was 200 ml. Three intraoperative complications occurred, including one severe skin emphysema with hypercapnia requiring an intensive care unit (ICU) admission, one bladder lesion occurring in a stage IIIB patient undergoing a laparoscopic RLH and radical colpectomy, and one obturator nerve lesion. No ICG injection-related complications were registered. Two major postoperative complications occurred: one small bowel perforation requiring a relaparotomy in a patient undergoing a full laparoscopic surgical staging, and a large bowel anastomosis breakdown requiring a relaparotomy in the patient undergoing a hemicolectomy.

**Fig. 1:**
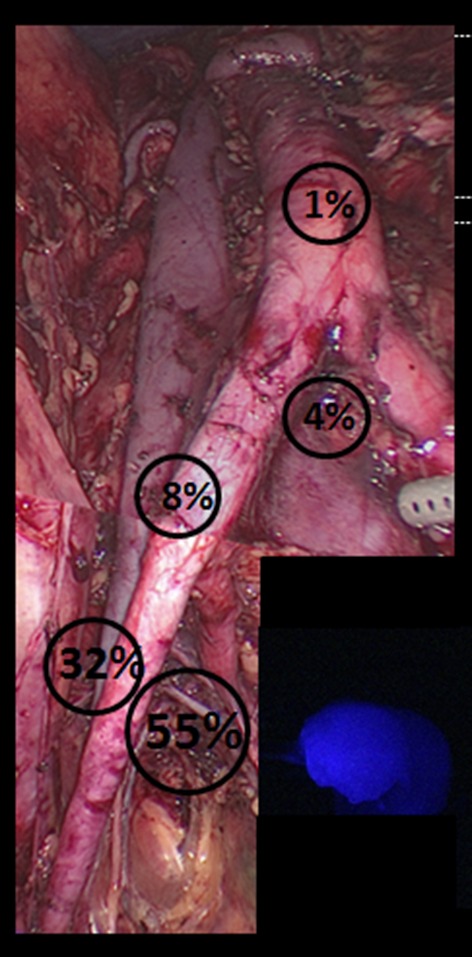
Anatomic distribution of sentinel lymph node. Obturator fossa 55 %, external iliac artery 32 %, common iliac artery 8 %, aortic bifurcation 4 %, para-aortic area 1 %

The median number of SLNs removed was three (range 0–11), and overall and bilateral SLN detection rates were 96 % (72/75) and 88 % (66/75), respectively. Data on SLN mapping are summarized in Table 2. In two of the three patients in whom the SLN mapping failed, the pelvis had been previously irradiated, secondary to bladder cancer and pelvic lymphoma, respectively. By excluding these patients from the analysis, these rates rise to 98.6 % (72/73) and 90.4 % (66/73), respectively. Every patient in whom at least one SLN could be identified mapped in the pelvis. In 5 % (4/75) of cases, additional SLNs were identified in the para-aortic region; no isolated para-aortic SLN was detected. The distribution of the SLNs is demonstrated in Fig. 1. Of the patients considered to be at low risk at frozen section, SLNs were metastatic in two cases.

**Table 2:** Lymph node data

Overall SLN detection	72/75 (96.0)
Bilateral SLN detection	66/75 (88.0)
Unilateral SLN detection	6/75 (8.0)
No SLN detection	3/75 (4.0)
Median number of SLNs/patient (range)	3 (0–11)
Patients with lymph node metastasis	11/75 (14.7)
Macrometastasis	7/11 (63.6)
Micrometastasis	4/11 (36.4)
Isolated tumor cells	0

Data in parentheses represent percentages unless otherwise stated

*SLN* sentinel lymph node

Overall, 14.7 % (11/75) of patients had lymph node metastases and, in all these cases, an SLN was detected bilaterally. Pathological evaluation of the SLNs showed micrometastases in four patients, all of which were diagnosed at H&E. Of the 42 patients in whom a PLND/PPALND was performed, one FN mapping occurred: both the bilateral SLNs in the pelvis and all the pelvic lymph nodes were negative, whereas two para-aortic NSLNs had metastatic disease. In another patient, four of five SLNs were true positive in the right hemipelvis, whereas one SLN in the left pelvis was FN as one NSLN had metastatic disease. The calculated sensitivity of the SLN mapping was 91.7 %; the FN rate was 8.3 %.

Thirty-three patients underwent a TLH/BSO and SLN biopsy, whereas 39 patients underwent the same surgical procedures followed by a PLND/PPALND. EBL and OR time were significantly lower among patients undergoing an SLN mapping only compared with patients undergoing a full lymphadenectomy (200 ml vs. 300 ml, *p* < 0.0001; 150 min vs. 300 min, *p* < 0.0001), but no differences were observed among intra- and postoperative complications in the two groups.

## Discussion

Although two randomized trials failed to show any benefit in EMCA patients who underwent a retroperitoneal staging, lymph node assessment still represents the cornerstone of EMCA staging.^19^,^20^ Lymph node status still represents one of the most important prognostic factors based on which adjuvant treatment is tailored.^21^–^24^

Our overall and bilateral detection rates of 96 % (72/75) and 88 % (66/75), respectively, are among the highest reported in the literature. Furthermore, by excluding two patients who had been previously subjected to pelvic radiation, these rates rise to 98.6 % (72/73) and 90.4 % (66/73), respectively. We speculate that the radiation-induced fibrosis alters the lymphatic flow, thus interfering with the SLN mapping; therefore, pelvic radiation should be a contraindication to SLN mapping. Similarly, Plante et al. reported overall and bilateral detection rates of 96 and 88 %, with a negative predictive value of 98.7 % per side in 50 patients with uterine malignancies undergoing a laparoscopic SLN mapping.^15^ More recently, Buda et al. compared the outcome of two different SLN mapping tracers in 81 women with uterine malignancies undergoing laparotomy or laparoscopic surgery.^17^ They reported higher overall and bilateral detection rates in patients undergoing SLN mapping with ICG compared with methylene blue. Their overall and bilateral detection rates and negative predictive value were 100, 88 and 100 %, respectively, when ICG was used. This is in accordance with our previously published data in cervical cancer.^14^

We believe that the good results recorded in our series are largely attributed to the tracer used. Desai et al. reported an overall and bilateral detection rate of 86 and 52 %, respectively, using blue dye,^10^ while in the SENTI-ENDO study, overall and bilateral detection rates were 89 and 69 %, respectively, using a combination of ^99^Tc and blue dye.^11^ Solima et al. reported an overall detection rate of 95 % after hysteroscopic injection of ^99^Tc; no data on their bilateral detection rate are reported.^25^ With the uterus being a midline structure, it is crucial to have a high bilateral detection rate to avoid unilateral lymph node dissections. Barlin et al. of the Memorial Sloan Kettering Cancer Center (MSKCC), proposed an SLN mapping algorithm for EMCA in which, in addition to the SLNs, every suspected lymph node is biopsied and a monolateral PLND is performed in those patients who map in only one hemipelvis.^26^ A high bilateral detection rate leads to a reduced number of bilateral and unilateral lymphadenectomies, possibly reducing surgical morbidity. By applying the MSKCC SLN mapping algorithm to our cohort of patients, one and six patients would have undergone a bilateral and monolateral PLND, respectively.

When SLN mapping is performed with ICG and the robotic platform, overall and bilateral detection rates vary between 87 and 82 and 97 and 57 % in the different series, respectively.^27^–^31^ Our results correlate well with these data, suggesting that the dye, rather than the robot, is accountable for it.

In our study, one patient with a stage IIIB EMCA underwent SLN mapping followed by full surgical staging. Although SLN mapping also proved to be reliable in this case, with the bilateral identification of true negative SLNs we do not recommend relying on the SLN biopsy only in patients with stages other than stage I.

Compared to other mapping protocols, we injected a higher concentration of ICG. According to a recent meta-analysis, a lower concentration of ICG may provide better performance of the SLN mapping.^32^ In our hands, our protocol worked well and in no case was the operative field excessively stained.

In our study, every patient in whom at least one SLN could be identified mapped in the pelvis. In 5 % (4/75) of cases, additional SLNs were identified in the para-aortic region. No isolated para-aortic SLNs were detected. Although the cervical injection of the tracer is the most common injection site in EMCA SLN mapping, there are concerns that tumors located in the fundus may spread through other lymphatic routes directly to the para-aortic region. For this reason, some centers propose a hysteroscopic tracer injection.^31^,^33^ In our series, only one case presented an isolated para-aortic lymph node metastasis, representing 2.4 % of the staged patients. This percentage is in accordance with the risk of isolated para-aortic metastasis reported by Mariani et al. in fully staged, high-risk EMCA patients, providing further evidence that cervical injection of the tracer represents a safe way to perform SLN mapping in EMCA.^34^

Finally, we demonstrated that SLN biopsy is associated with a shorter OR time and a lower EBL compared with full surgical staging. Although these results may seem trivial, they have not yet been reported in the literature. However, our unique cohort of patients, including those who have undergone a SLN biopsy only and patients who have undergone SLN biopsy followed by a full lymphadenectomy, allowed for such an analysis. Intra- and postoperative complication rates are low and do not differ between the two groups. These results confirm data from the literature showing that, in experienced hands, a full laparoscopic staging is a feasible procedure, with low complication rates.^35^ The acquisition of the skills to perform a full pelvic and para-aortic laparoscopic lymphadenectomy is long and steep.^36^ Although SLN mapping seems to be more intuitive and require less surgical skill, it is crucial that it is performed accurately in order to avoid running the risk of sampling the wrong lymph node. Furthermore, although it is debated whether or not a full lymphadenectomy should be performed in patients with metastatic SLNs, considering that the MSKCC SLN mapping algorithm should be applied, we believe that every physician who performs a laparoscopic SLN mapping should have the skills to perform a full retroperitoneal staging.

Limitations of the study include its retrospective nature and the relatively small sample size. On the other hand, the prospective SLN mapping data collection, the relatively large percentage of patients undergoing PLND/PALND, and the thoroughness of the lymphadenectomy, as testified by the large number of lymph nodes removed, signify the strengths of the study.

## Conclusions

This is the largest existing series on laparoscopic ICG SLN mapping in EMCA. Laparoscopic ICG SLN mapping in EMCA is characterized by high overall and bilateral detection rates and low FN rates that compare favorably with those reported when a robotic platform is used, suggesting that the tracer, rather than the surgical approach, is responsible for the SLN mapping outcome.

Compared with full surgical staging, SLN biopsy is associated with significantly lower EBL and shorter OR time, but no difference in intra- and postoperative complications. In a dualistic model in which a lymphadenectomy is fully performed or omitted based on intraoperatively evaluated pathologic tumor characteristics, ICG SLN mapping appears to represent a solomonic solution that gathers important pathologic information at low surgical expense.
